# Cloning and Functional Characterization of Two Germacrene A Oxidases Isolated from *Xanthium sibiricum*

**DOI:** 10.3390/molecules27103322

**Published:** 2022-05-22

**Authors:** Dong-Mei Xie, Qiang Zhang, Ling-Kai Xin, Guo-Kai Wang, Cong-Bin Liu, Min-Jian Qin

**Affiliations:** 1 Department of Resources Science of Traditional Chinese Medicines, China Pharmaceutical University, Nanjing 210009, China; xiedongmei@ahtcm.edu.cn; 2 Anhui Province Key Laboratory of Research & Development of Chinese Medicine, Anhui University of Chinese Medicine, Hefei 230012, China; zhangqiang@stu.ahtcm.edu.cn (Q.Z.); xinglingkai@stu.ahtcm.edu.cn (L.-K.X.); wanggk@ahtcm.edu.cn (G.-K.W.)

**Keywords:** *Xanthium sibiricum*, sesquiterpene lactones, biosynthesis, functional analysis, germacrene A

## Abstract

Sesquiterpene lactones (STLs) from the cocklebur *Xanthium sibiricum* exhibit significant anti-tumor activity. Although germacrene A oxidase (GAO), which catalyzes the production of Germacrene A acid (GAA) from germacrene A, an important precursor of germacrene-type STLs, has been reported, the remaining GAOs corresponding to various STLs’ biosynthesis pathways remain unidentified. In this study, 68,199 unigenes were studied in a de novo transcriptome assembly of *X. sibiricum* fruits. By comparison with previously published GAO sequences, two candidate *X. sibiricum* GAO gene sequences, *Xs*GAO1 (1467 bp) and *Xs*GAO2 (1527 bp), were identified, cloned, and predicted to encode 488 and 508 amino acids, respectively. Their protein structure, motifs, sequence similarity, and phylogenetic position were similar to those of other GAO proteins. They were most strongly expressed in fruits, according to a quantitative real-time polymerase chain reaction (qRT-PCR), and both *Xs*GAO proteins were localized in the mitochondria of tobacco leaf epidermal cells. The two *Xs*GAO genes were cloned into the expression vector for eukaryotic expression in *Saccharomyces cerevisiae*, and the enzyme reaction products were detected by gas chromatography–mass spectrometry (GC-MS) and liquid chromatography–mass spectrometry (LC-MS) methods. The results indicated that both *Xs*GAO1 and *Xs*GAO2 catalyzed the two-step conversion of germacrene A (GA) to GAA, meaning they are unlike classical GAO enzymes, which catalyze a three-step conversion of GA to GAA. This cloning and functional study of two GAO genes from *X. sibiricum* provides a useful basis for further elucidation of the STL biosynthesis pathway in *X. sibiricum*.

## 1. Introduction

Sesquiterpene lactones (STLs) are widely distributed in nature and have a broad range of beneficial biological activities, including anti-bacterial, anti-inflammatory, and anti-cancer effects [1,2,3,4,5]. Two specific STLs, xanthatin and xanthinosin, are produced in the burs and leaves of *Xanthium* L. plants [6,7,8]. Many studies have been conducted on the quality and pharmacological activities of *X. sibiricum*. However, the details of the biological pathways associated with the anti-cancer effects of STLs in *Xanthium* species remain unclear.

**Figure 1:**
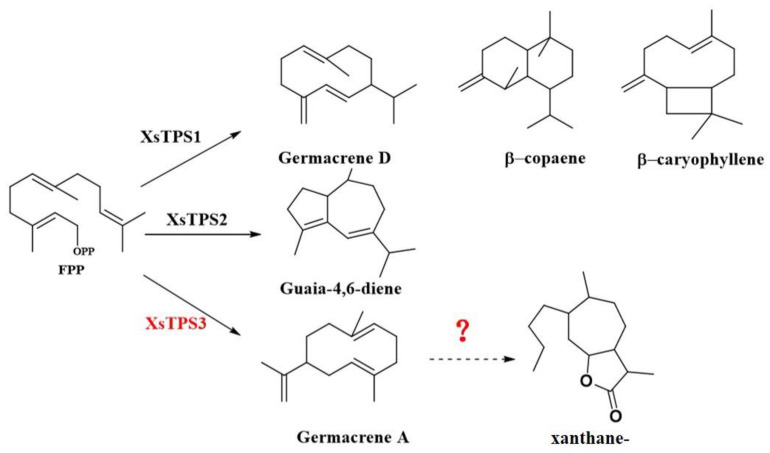
Overview of STL synthesis in *X. stramonium*. Solid lines are actual biosynthetic steps, and the dashed line indicates the hypothesized synthetic step.

Based on the carbon skeleton, STLs can be classified into multiple types, including germacrene, guaiane, xanthane, pseudoguaiane, eudesmane, and elemane lactones [9]. The molecular mechanisms of STLs differ among types. For example, eudesmane-type STLs synthesize the core carbon skeleton 10-epi-junenol before lactone ring synthesis [10], while guaiane-type STLs are produced from germacrene-type STLs and are induced by protonation [11]. The STL synthesis pathway is usually divided into three main processes: The first process is the synthesis of intermediates, including isopentenyl pyrophosphate (IPP) and dimethylallyl pyrophosphate (DMAPP). The second step involves the formation of the sesquiterpene skeleton, which is preceded by the formation of farnesyl pyrophosphate (FPP) from IPP and DMAPP. Sesquiterpene synthase (STP) then catalyzes FPP to produce the sesquiterpene skeleton. The final step is the formation of the STL end-product, which involves a variety of structural modifications to the carbon skeleton. In particular, STP is critical for the structural transformation of FPP to STL, which subsequently catalyzes the formation of multiple types of sesquiterpenes through a series of chemical processes such as intermediate cyclization of carbenium ion, deprotonation, and hydrogen transfer [12,13,14,15,16,17]. Importantly, some STPs, the cytochrome P450 enzymes, play a modulatory role in SLT biosynthesis, participating in the addition of functional groups to the sesquiterpene backbone [18]. For example, the cytochrome P450 GAO, isolated from an Asteraceae plant, catalyzes the three-step sequential oxidation of germacrene A to GAA [19]. In addition, in common chicory (*Cichorium intybus* L.), the most critical modification enzyme in the biosynthetic pathway of the 6α-type STL-myrcene lactone C12 is the cytochrome P450 enzyme [20]. It was also shown that the cytochrome P450 enzymes parthenolide synthase (PTS) and kauniolide synthase (KLS), cloned from the aster *Chrysanthemum paludosum*, catalyzed the oxidation reaction of the C4–5 double-bonds of costunolide, which in turn, generated parthenolide [11,21]. Since germacrene-derived STLs are the simplest, a number of studies have investigated these STLs using synthetic biology and related techniques. Three STP genes were cloned from *X. strumarium*: *XS*TPSS1 catalyzed the production of germacrene D, *XS*TPSS2 catalyzed the formation of Guaia-4,6-diene, and *XS*TPSS3 catalyzed the production of germacrene A (Figure 1) [22]. However, it is not clear how different biologically active STLs are produced in *Xanthium* species after sesquiterpene skeleton formation.

**Figure 2:**
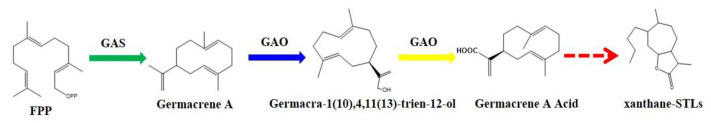
Hypothesized STL biosynthetic pathway in *X. sibiricum*.

Studies have shown that GAA is an important precursor substance in the biosynthesis pathway of germacrene-derived STLs [19]. Based on the findings outlined above, we hypothesized that, in *X. sibiricum*, GAO would catalyze the production of GAA from germacrene A to produce GAA in two consecutive steps (Figure 2).

To test this hypothesis, we identified the genes homologous to GAO by searching known GAO gene sequences in the National Center for Biotechnology Information (NCBI) database against a transcriptome library of *X. sibiricum* established therein and cloned the GAO gene using complementary DNA (cDNA). Subsequently, bioinformatic analyses of the predicted amino acid and protein structures, gene expression patterns, and subcellular localization were carried out. The enzyme reaction products were detected by GC-MS and LC-MS methods, where GAO genes were cloned into the expression vector for eukaryotic expression in *S. cerevisiae*. The results clarify the downstream STL synthesis pathway in *X. sibiricum* in future work.

## 2. Results

### 2.1. Establishment of a Transcriptome Library and Gene Annotation of X. sibiricum

The de novo transcriptome library of *X. sibiricum* included 5,989,562,311 nucleotides (nt), and the transcriptome Q20, N, and GC percentages were 97.57%, 0.01%, and 45.36%, respectively. After low-quality reads and filtering out those containing duplicates or junctions, 49,957,916 valid clean reads remained. The clean reads were assembled de novo using Trinity assembly software [23], and a total of 68,199 unigenes were obtained, with an average length of 639 nt and an N50 of 954 nt.

**Figure A1:**
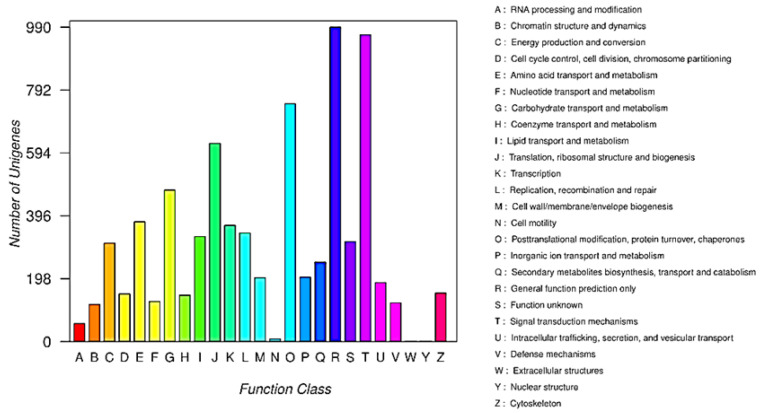
COG classification of *X. sibiricum* unigenes.

Protein function annotation information for all unigenes was obtained using BLAST [24]. Of the 68,199 unigenes, 19,129, 13,845, 24,721, 37,205, and 14,001 unigenes were successfully annotated with the Pfam, Kyoto Encyclopedia of Genes and Genome (KEGG), SwissProt, non-redundant protein sequence database (NR), and string libraries, respectively. A total of 13,619 unigenes from the transcriptome were successfully annotated to the Cluster of Orthologous Groups of proteins (COG) database, corresponding to the 25 functional categories. A total of 990 unigenes mainly focused on function prediction, with the highest percentage focusing on STLs, and a further 250 unigenes were annotated to secondary metabolite biosynthesis, transport, and catabolism (Appendix A, Figure A1).

**Figure A2:**
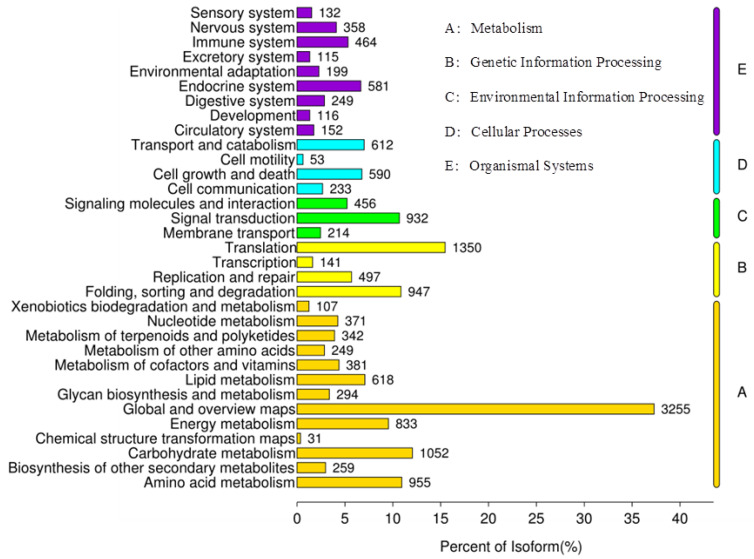
Pathway analysis of associated *X. sibiricum* unigenes in KEGG.

A total of 13,845 unigenes were annotated to 128 metabolic pathways in the KEGG database, and 342 were annotated to “metabolism of terpenoids and polyketides” (Appendix A, Figure A2). Among these 342 unigenes, 89 were related to “terpenoid backbone biosynthesis”, and 22 were involved in “sesquiterpenoid and triterpenoid biosynthesis.”

In plants, the biosynthesis of STLs primarily occurs through the mevalonate (MVA) or methyl-D-erythritol phosphate (MEP) pathways, which synthesize DMAPP and IPP precursors [14]. KEGG pathway analysis showed that a total of 31 transcripts in *X. sibiricum* encoded six enzymes of the MVA pathway (acetyl-CoA C-acetyltransferase [ACCT, E.C.No:2.3.1.9], 3-hydroxy-3-methylglutaryl coenzyme A synthetase [HMGS, E.C.No:2.3.1.10], 3-hydroxy-3-methylglutaryl coenzyme A reductase [HMGR, E.C.No:1.1.3.34], mitogen-activated protein kinase/extracellular signal-regulated kinase [MEK, E.C.No:2.7.1.36], phosphomevalonate kinase [PMK, E.C.No:2.7.4.2], and pyrophospomevalonate decarboxylase [MVD, E.C.No:4.1.1.33]), and 21 transcripts encoded seven enzymes in the MEP pathway (1-deoxy-D-xylulose-5-phosphate synthase [DXS, E.C.No:2.2.1.7], 1-deoxy-D-xylulose 5-phosphate reductoisomerase [DXR, E.C.No:1.1.1.26], 2-C-methyl-D-erythritol 4-phosphate cytidyltransferase [MCT, E.C.No:2.7.7.60], 4-diphosphocytidyl-2-C-methyl-D-erythritol kinase [CMK, E.C.No:2.7.7.148], 2-C-methyl-D-erythritol 2,4-cyclodiphosphate synthase [MDS, E.C.No:4.6.1.12], 4-hydroxy-3-methylbut-2-en-1-yl diphosphate synthase [HDS, E.C.No:1.17.7.1], and 4-hydroxy-3-methylbut-2-enyl-diposphate reductase [HDR, E.C.No:1.17.1.2]).

### 2.2. Cloning and Bioinformatics Analysis of XsGAO Genes

**Figure A3:**
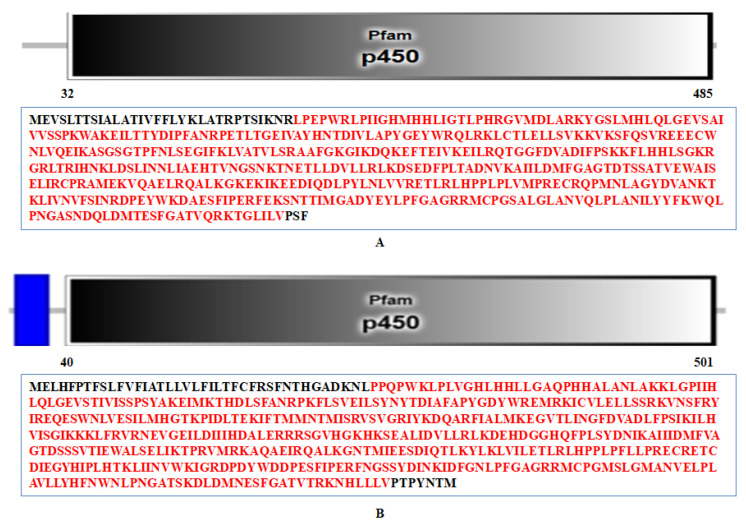
Domain prediction and amino acid sequences of *Xs*GAO1 (**A**) and *Xs*GAO2 (**B**). (Function domains shown in red).

**Figure A4:**
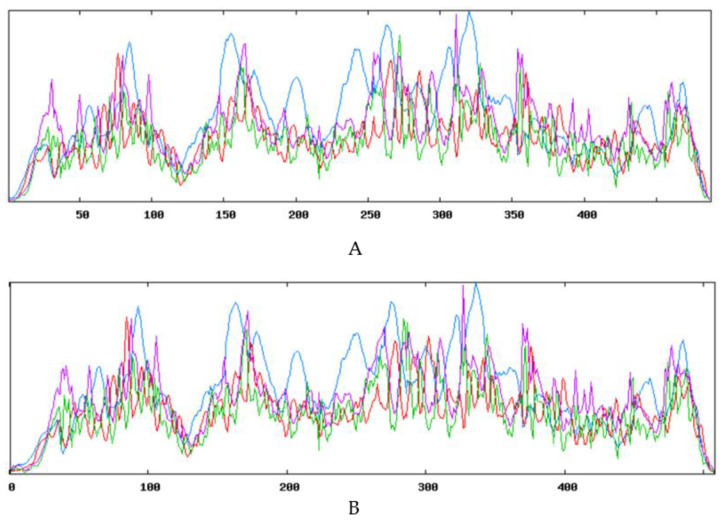
Secondary structures of (**A**) *Xs*GAO1 and (**B**) *Xs*GAO2. α-helixes are indicated in blue, β-sheets are indicated in green, and random coils are indicated in red.

**Figure A5:**
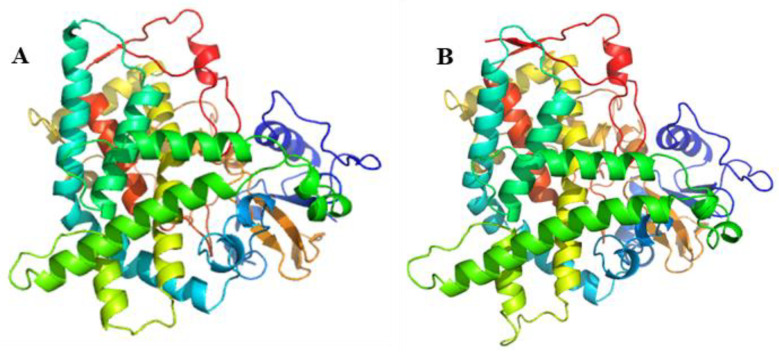
Predicted 3D structures of (**A**) *Xs*GAO1 and (**B**) *Xs*GAO2.

Two candidate GAO genes were designed using these sequences as templates (Appendix A, Table A1). The full lengths of *Xs*GAO1 and *Xs*GAO2 were 1467 and 1527 bp, encoding 488 and 508 amino acids, respectively. SMART (Simple Modular Architecture Research Tool) analysis showed that *Xs*GAO1 and *Xs*GAO2 encoded proteins with molecular weights of approximately 54.98 and 58.01 kDa, respectively, protein isoelectric points of approximately 8.72 and 8.40, respectively, and protein a pH value of 5.1. Protein domain analysis showed that *Xs*GAO1 harbored a P450 domain comprising 454 amino acids (amino acid 42-485, Appendix A, Figure A3A,) and *Xs*GAO2 harbored a P450 domain comprising 462 amino acids (amino acid 40-501, Appendix A, Figure A3B). The secondary structures of the *Xs*GAO1 and *Xs*GAO2 proteins were predicted using SOMPA online. The prediction of *Xs*GAO1 indicated that 244 amino acid residues (50%) were involved in the formation of α-helix, 66 residues (13.52%) were involved in the extended chain, 27 residues (5.53%) were involved in *β*-turn, and 151 residues (30.94%) were involved in a random coil (Appendix A, Figure A4A). Prediction of *Xs*GAO2 indicated that 253 amino acid residues (49.8%) were involved in the formation of α-helix, 62 residues (12.2%) were involved in the extended chain, 31 residues (6.1%) were involved in *β*-turn, and 162 residues (31.89%) were involved in a random coil (Appendix A, Figure A4B). To better characterize the *Xs*GAO bioinformation, 3D-structure prediction of the *Xs*GAO1- and *Xs*GAO2-encoded proteins was performed, and spatial predictions showed high similarity with the P450 enzyme from *Salvia miltiorrhiza* (Appendix A, Figure A5). 

**Figure 3:**
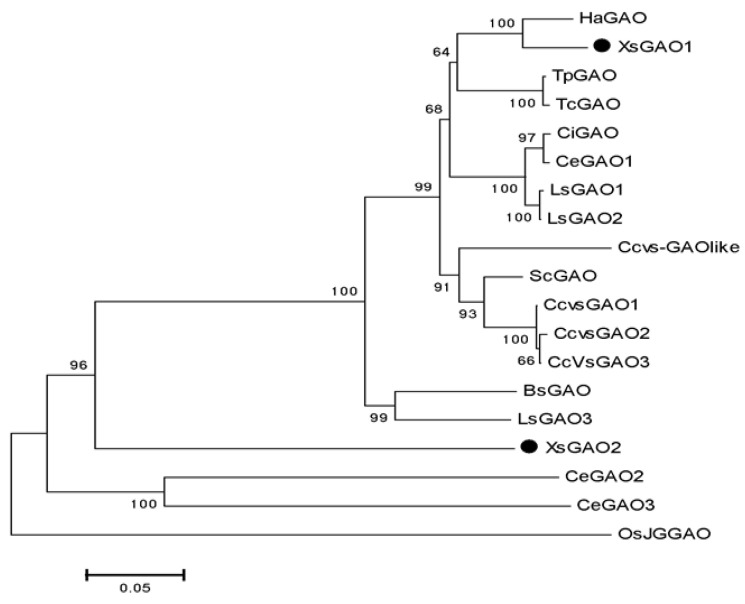
Phylogenetic tree analysis of GAOs is shown. *OsJG*GAO used as outgroups, which is from *Oryza sativa* Japonica Group. Bootstrap value were shown in percentage values from 1000 replicates.

BLAST analysis showed that *Xs*GAO1 and *Xs*GAO2 were highly similar to 17 GAO protein homologs from 10 species, which were obtained from the NCBI database (Appendix A, Table A3). Phylogenetic tree analysis indicated that two *Xs*GAO genes were mainly conserved in Asteraceae; the amino acid sequence of *Xs*GAO1 shared 94.06% sequence identity with *Ha*GAO from *Helianthus annuus* (XP_022000663.1), while *Xs*GAO2 was recovered on a distinct branch (Figure 3).

### 2.3. XsGAO1 and XsGAO2 Expression Patterns in X. sibiricum

**Figure 4:**
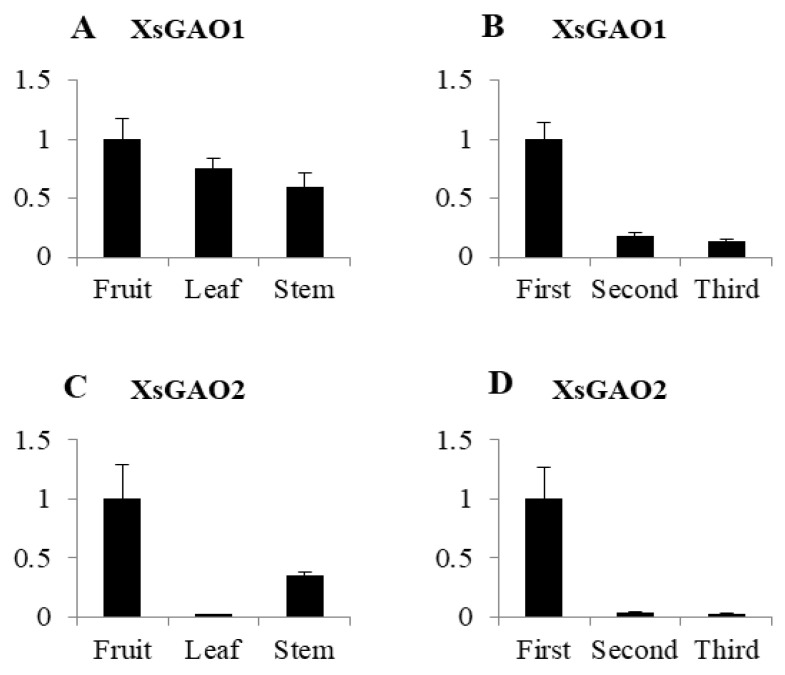
Expression of *Xs*GAO1 and *Xs*GAO2 in different organizations and periods ((**A**,**C**) are the expressions in different organs; (**B**,**D**) are the expressions at different times).

Across the three *X. sibiricum* tissues tested (fruit, leaves, and stems), the expression patterns of *Xs*GAO1 and *Xs*GAO2 were similar: both genes were more strongly expressed in the fruits than the leaves or stems (Figure 4A,C). 

**Figure 5:**
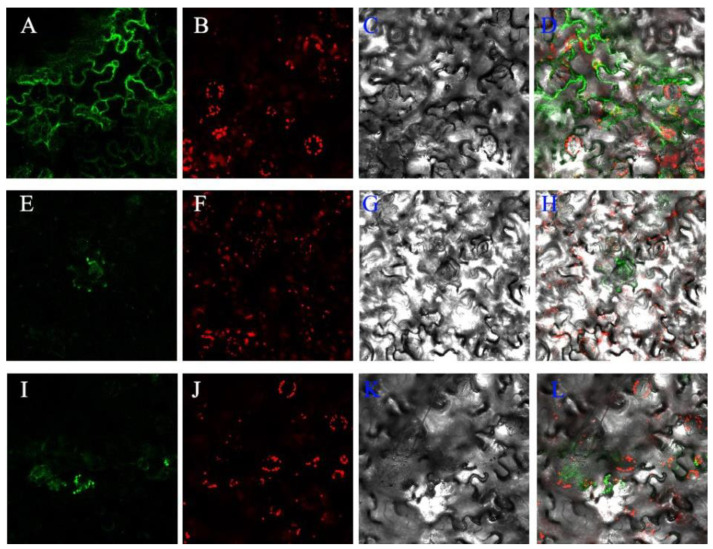
Subcellular localization of *Xs*GAO1 and *Xs*GAO2 fusion proteins in tobacco leaves. Images show tobacco leaves transformed with (**A**–**D**) the 1300-GFP empty plasmid, (**E**–**H**) the 35S: GAO1-GFP plasmid, and (**I**–**L**) the 35S: GAO2-GFP plasmid under various lights. Green signals correspond to the target gene fused with green fluorescent protein (GFP) after excitation at 488 nm; red signals correspond to the chloroplasts’ autofluorescence after excitation at 488 nm.

To localize *Xs*GAO1 and *Xs*GAO2 in the cell, tobacco leaf transformation was performed. Confocal laser scanning microscopy (CLSM) examination of the transformed tobacco leaves identified *Xs*GAO1 and *Xs*GAO2 signals in the mitochondria (Figure 5). This was consistent with predictions based on their sequence features.

### 2.4. Functional Study of XsGAO1 and XsGAO2

**Figure A6:**
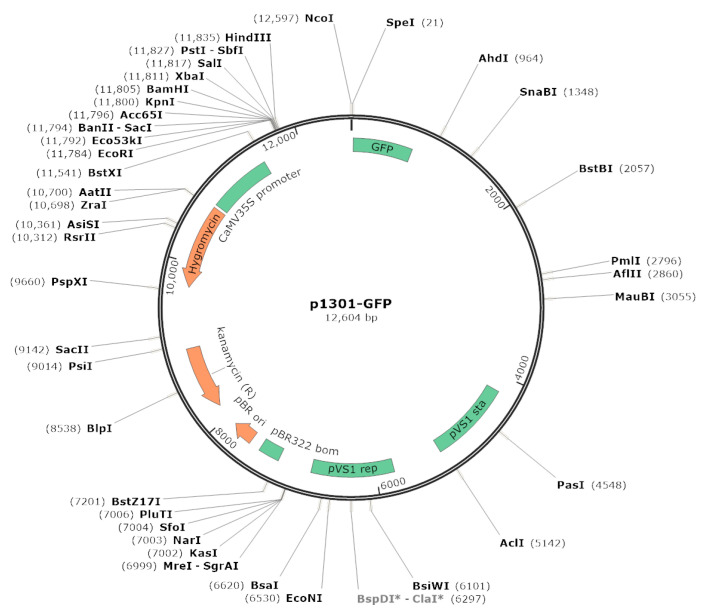
Map of the vector used for subcellular localization of *Xs*GAO1 and *Xs*GAO2 created with SnapGene. BspDI* and ClaI* are isoschizomers.

**Figure A7:**
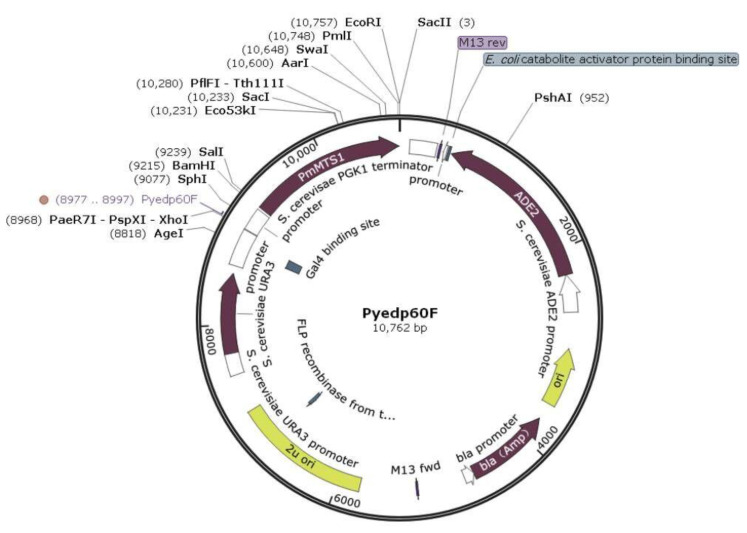
Map of the vector used in the yeast expression experiment created with SnapGene. Different protein showed with distinguished color.

**Figure A8:**
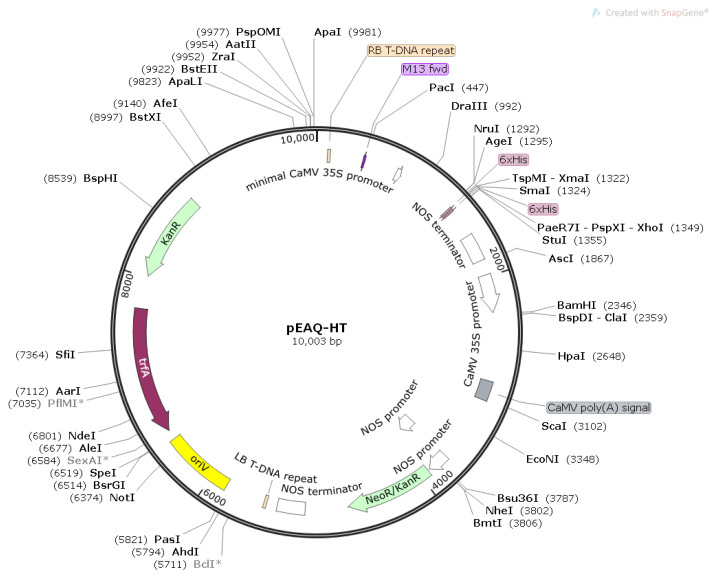
Map of the vector used for function validation of *Xs*GAO1 and *Xs*GAO2 in tobacco created with SnapGene. BclI* is methylation sensitive restriction endonuclease. Different protein showed with distinguished color.

**Figure A9:**
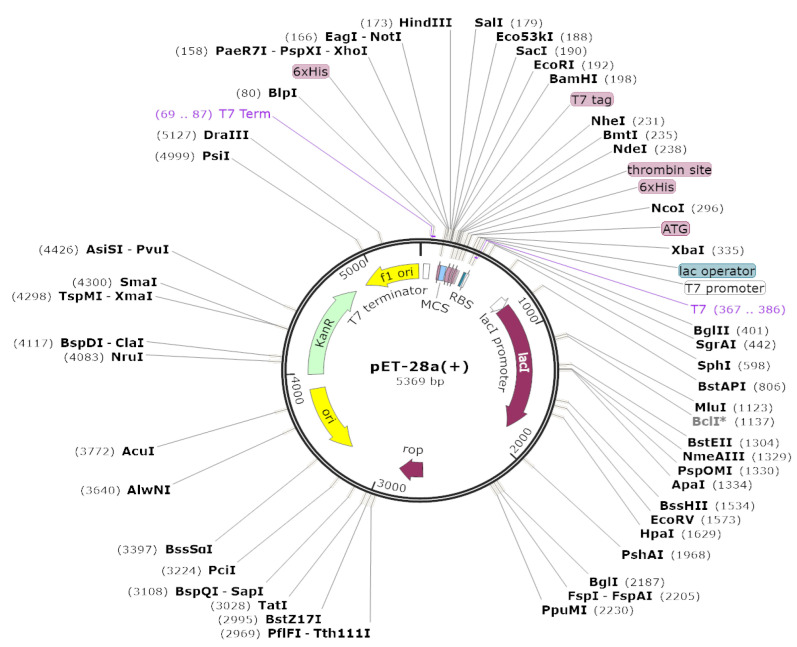
Map of the vector used for prokaryotic expression created with SnapGene. Different protein showed with distinguished color.

The full-length *Xs*GAO genes were cloned into the yeast expression vector pYeDP60 and co-transferred into *S. cerevisiae* WAT11. Compared with the expression of *Ls*GAS alone or the control yeast bearing the empty vector, germacrene A was obtained through prokaryotic expression and recombinant protein enzyme activity assays and was used as the substrate for *Xs*GAO1 and *Xs*GAO2 in the enzymatic activity reaction. Additionally, the *Ls*GAO gene was cloned from *Lactuca sativa*, and the microsomal protein expressed in this gene was used as a positive control. The inactivated microsomes were used as a negative control to verify whether the microsomal protein expressed in *Xs*GAO1 and *Xs*GAO2 showed catalytic activity through a two-step enzyme activity catalytic assay (Appendix A, Figure A6, Figure A7, Figure A8 and Figure A9).

**Figure 6:**
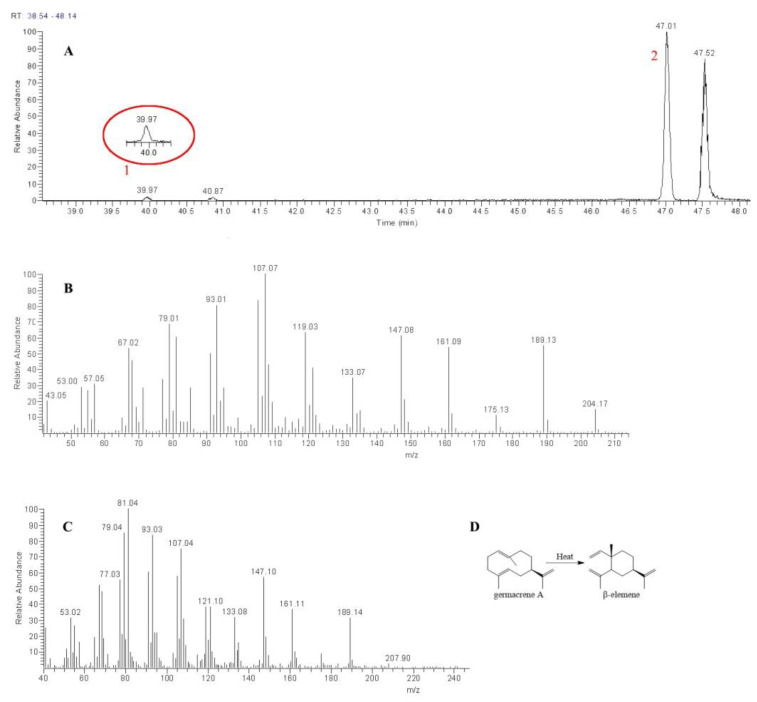
*Ls*GAS gene catalyzed the production of GA from FPP. (**A**) A GC-MS ion chromatogram, in which peak 1 corresponds to *β*-elemene and peak 2 corresponds to GA. (**B**) Fragment ion of GA. (**C**) Fragment ion of *β*-elemene. (**D**) Rearrangement of germacrene A to *β*-elemene through the application of heat.

The gas chromatography–mass spectrometry (GC-MS) chromatogram of enzymatic activity experiments on the *Ls*GAS recombinant protein showed two distinct peaks (Figure 6A). The second peak exhibited fragment ion peaks at *m*/*z* 53, 67, 79, 93, 107, 119, 133, 147, 161, 175, 189, and 204 (Figure 6B), which corresponded to the characteristic ions of germacrene A. However, the first peak exhibited fragment ion peaks at *m*/*z* 77, 81, 93, 107, 121, 133, 147, 161, and 189, which corresponded to the characteristic ions of *β*-elemene (Figure 6C) [10,25]. Therefore, we assumed that peak 1 corresponded to *β*-elemene and peak 2 corresponded to germacrene A.

**Figure 7:**
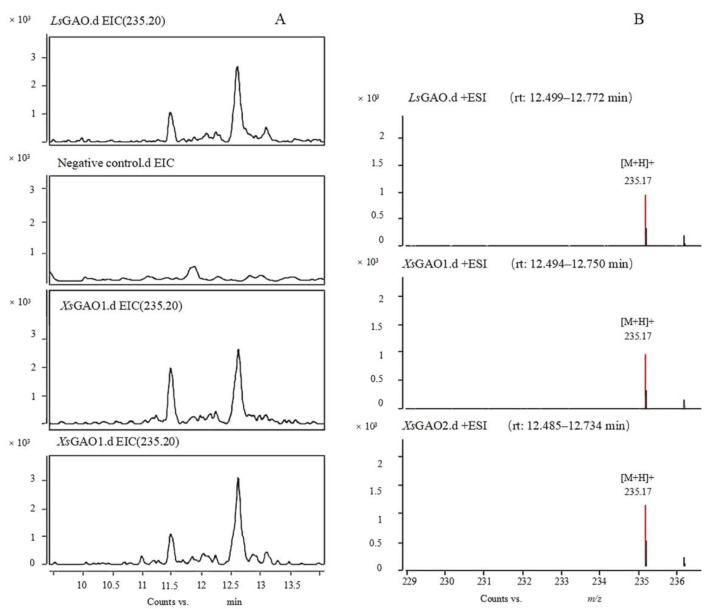
*Ls*GAO, *Xs*GAO1, and *Xs*GAO2 catalyzed germacrene A. (**A**) Renal chromatogram and (**B**) mass spectrum.

An analysis of the enzymatic activity of *Xs*GAO1 and *Xs*GAO2 in microsomes identified peaks in both liquid chromatography–mass spectrometry (LC-MS) chromatograms with the same retention time as the positive control *L*sGAO (tR = 12.54 min; Figure 7A). Further analysis showed that the peaks produced in all three assays (i.e., *Xs*GAO1, *Xs*GAO2, and *L*sGAO) had the same *m*/*z* in the positive ion mode ([M+H]^+^ = 235.17; Figure 7B), suggesting that this peak corresponded to GAA. The results indicated that the *Xs*GAO1 and *Xs*GAO2 proteins both catalyzed the production of GAA from germacrene A in yeast microsomes.

## 3. Discussion

To investigate plant gene expression and analyze its function, transcriptome sequencing is an important molecular method that can provide genetic information in the absence of genomic data [26]. The de novo assembly platform greatly contributes to finding new genes, providing databases of sesquiterpene synthases and cytochrome P450s for cloning in *X. strumarium* [27]. *X. sibiricum* is a traditional plant containing unique secondary metabolites, of which STLs have various pharmaceutical properties. Although sesquiterpene synthase (STP) has only been cloned from *X. strumarium* glandular trichomes [28], transcriptome databases established from fruits of *X. sibiricum* provided cDNAs of two GAO genes that were cloned accurately in this study. 

As an enzyme that produces an important precursor substance for the synthesis of STLs, the GAO gene is conserved in Asteraceae [25,29]. To investigate the function of this gene, the full-length cDNA sequences of *Xs*GAO1 and *Xs*GAO2 were successfully cloned. Phylogenetic analysis indicated that *Xs*GAO1 may have a similar function to the GAO in *H. annuus*. However, *Xs*GAO2 was distinct from other GAOs, representing a separate branch that needs further investigation. Multiple comparisons showed that the predicted *Xs*GAO1 protein had high homology with other redox-like proteins, and its protein sequence contained conserved amino acid residues that are expected in the cytochrome P450 enzyme family [30]. Analysis of the 3D structure predicted that *Xs*GAO1 and *Xs*GAO2 had functions similar to those corresponding with ferruginol synthase (CYP71 family).

The expression patterns of *Xs*GAO1 and *Xs*GAO2 in *X. sibiricum* leaves differed over time, with the highest expression level observed in young leaves, and expression levels decreasing with maturation. *Xs*GAO1 and *Xs*GAO2 were also differentially expressed among fruits and stems, presumably related to their functions. The expression of the two *Xs*GAOs was the highest in fruits, which explains why fruits with higher contents of STLs are used in the traditional Chinese medicine Cang Er Zi.

*Xs*GAO1 and *Xs*GAO2 cDNAs with the correct sequence were successfully inserted into an expression vector and used in transient transformation assays of *Nicotiana benthamiana*. However, no fluorescence was observed in the protoplasts. This may be because the accumulated concentration of the product was below the detection limit of the instrument or because the GAA generated was intermediately transient in *N. benthamiana* [31]. Both *Xs*GAO1 and *Xs*GAO2 were localized in the mitochondria, which was consistent with terpene synthase in tomatoes (also localized in the mitochondria) [32,33].

GAO isolated from *L. sativa* was expressed in an engineered yeast to synthesize GAA de novo, and the classical GAO activity involved three-step oxidation of germacrene A (GA) to yield GAA and 12,6-guaianolides [25,34], similar to *N. benthamiana* [35]. Meanwhile, an *Xs*GAO from *X. strumarium* catalyzed only one-step conversion of germacrene A to germacrene alcohol [36], but this study clearly shows that *Xs*GAO1 and *Xs*GAO2 catalyzed a second step of oxidation of the non-natural substrate germacrene A to germacrene A acid, which was not observed in yeast with a different GAO. Apparently, *Xs*GAO2 is a unique enzyme, a functional adaption of SLTs’ biosynthetic pathway diversification. Of course, a structural analysis of the *Xs*GAO2 biochemical function, such as to identify the active center and crystal structure of oxidase, would help to examine whether it has unique GAO activity. In addition, with the advent of CRISPR-Cas genome editing, CRISPR-Cas-mediated gene knockout in tomatoes and the medicinal plant *Salvia miltiorrhiza* has been successfully performed [37,38]. This approach could be applied to verify the function of *Xs*GAO2 in the future.

Beyond this, *X. sibiricum* contains a variety of biologically active STLs, mainly xanthane-STLs with anti-tumor activities [39,40]. As such, studying the genes in the STL synthesis pathway could provide new ideas for the investigation of xanthane-STL biosynthesis pathways.

## 4. Materials and Methods

### 4.1. Establishment of a Transcriptome Library and Gene Annotation

The fresh samples (fruit, leaf, and stem) of *X. sibiricum* used for the RNA extraction were collected from Chaoyang District (N: 40.0031, E: 116.5468, H: 113 m, Beijing, China) in August, wrapped in tinfoil, and frozen immediately in liquid nitrogen for storage at −80 °C. Species verification was performed by Professor Dongmei Xie at the School of Pharmacy (Anhui University of Chinese Medicine). The total ribonucleic acid (RNA) was isolated using a TransZol Up Plus RNA kit (*TansGen* Biotech, Beijing, China), according to the manufacturer’s protocol. The RNA extract was reverse-transcribed and then sequenced on an Illumina HiSeq 3000 platform at Shanghai Majorbio Bio-pharm Technology Corporation. After high-throughput sequencing, unigenes were assembled de novo from the clean reads obtained from the raw sequencing reads. To predict the biological function, all unigenes were annotated via a similarity search against the public databases, which contained Pfam, NR (http://www.ncbi.nlm.nih.gov/) (accessed on 5 May 2018), SwissProt (http://www.uniprot.org/) (accessed on 6 May 2018), KEGG (http://www.genome.jp/kegg/) (accessed on 8 May 2018), GO (Gene Ontology, http://www.geneontology.org/) (accessed on 10 May 2018), and BlastX (E value < 1 × 10^−5^) [24,26].

### 4.2. Cloning and Bioinformatics Analysis of XsGAO1 and XsGAO2

GAO gene sequences were searched for in the transcriptome database of *X. sibiricum* using local BLAST, with seed sequences downloaded from the NCBI. The full-length cDNA of two candidate genes, *Xs*GAO1 and *Xs*GAO2, was then cloned using reverse transcription polymerase chain reaction (RT-PCR) (primers are listed in Appendix A, Table A1). 

The nucleotide sequences and their encoded amino acid sequences were analyzed using bioinformatics software, and the physicochemical properties of the encoding proteins were predicted using vector NTI, open reading frames (ORF), and amino acid sequence translation through Expasy Translate (http://web.expasy.org/translate/) (accessed on 1 March 2020). Gene domain analysis was performed using SMART (http://smart.embl-heidelberg.de/) (accessed on 5 March 2020) [41].

Phylogenetic relationships were constructed using the amino acid sequences of *Xs*GAO1 and *Xs*GAO2 with different reported GAO sequences. Nineteen sequences were aligned using ClustalX2, and the alignment was used to construct a phylogenetic tree using MEGA5.0 software [42].

Secondary protein structures were obtained using SOMPA online analysis software (http://npsa-pbil.ibcp.fr/cgi-bin/npsa_automat.pl?page=npsa_sopma.html) (accessed on 20 March 2020), and 3D structures and peptide-sequence fragments of XsGAO proteins were predicted using the SWISS-MODEL web server (https://swissmodel.expasy.org/interactive) (accessed on 20 March 2020) and PyMOL software [43].

### 4.3. Examination of the Expression Patterns of the XsGAO1 and XsGAO2 Genes 

To further understand the distribution characteristics of GAO genes in *X. sibiricum*, qRT-PCR (primers are listed in Appendix A, Table A2) was employed to determine the expression patterns of two GAO genes in different organs (leaf, stem, and fruit) at different stages of *X. sibiricum*. RNA was extracted according to protocol provided for the TRlzol reagent (Invitrogen), and this was then converted into cDNA via reverse transcription with the TransScriptor First-Strand cDNA Synthesis Supermix kit. Subcellular localization of the protein was observed using CLSM, and the consistency with prediction was determined using SLP-Local (https://sunflower.kuicr.kyoto-u.ac.jp/~smatsuda/slplocal.html) (accessed on 25 March 2018) online [27,43].

### 4.4. Functional Study of XsGAO1 and XsGAO2 Genes in Yeast

The ORFs of *Xs*GAO1 and *Xs*GAO1 were PCR-amplified (primers are listed in Appendix A, Table A2), and the amplicons were digested with BamHI/EcoRI and cloned into the respective sites in pYeDP60-*Xs*GAO (pYeDp60 plasmid provided by the Department of Pharmacology, Second Military Medical University). To supply the substrate for *Xs*GAOs, the germacrene A synthase gene (*Ls*GAS; AF489965) from *L. sativa* (provided by the Department of Pharmacognosy, Second Military Medical University) was inserted into the *E. coli* expression vector pet28a-*Ls*GAS at EcoRI-SacI sites. Germacrene A was produced by *Ls*GAS, which was expressed through Transetta (DE3), and then germacrene A was catalyzed by the *Xs*GAO gene expressed in *S. cerevisiae* WAT11 (WAT11 provided by the China Academy of Chinese Medical Sciences). For comparison, we used a classical GAO from *L. sativa* (*Ls*GAO; GU198171) that is known to oxidize germacrene A in a three-step oxidation process. Transgenic yeast cells were cultivated in appropriate dropout media, and the expression of the transferred genes was induced by 2% galactose [25,34,41].

### 4.5. GC-MS and LC-MS Analyses

GC-MS analysis was performed [25] using a Shimadzu GC-MS TRACE GC Ultra/DSQ II instrument (Thermo Fisher Scientific, Waltham, MA, USA). A sample volume of 1 μL was injected at an inlet temperature of 150 °C, and compounds were separated using a DB-5MS column (30 m × 250 μm × 0.1 μm) with helium as a carrier gas at a flow rate of 2 mL/min. The GC oven temperature program was as follows: 45 °C for 4 min, 45–170 °C for 67 min, and 170 °C for 72 min. The electron impact ionization of the mass spectrometric detector was tuned to 70 eV and operated at 40–400 Da in full scan mode. LC-MS/MS analysis was performed [41] using a UHPLC-Q-TOF-MS system (Agilent Technologies, Santa Clara, CA, USA) equipped with an XBridge^TM^ C_18_ column (2.1 × 100 mm; Waters Corporation, Milford, MA, USA), with a mobile phase of 0.1% formic acid aqueous solution (A) and 0.1% acetonitrile (B) at a flow rate of 0.4 mL/min, injection volume of 1 μL, and column temperature of 40 °C. Mass spectrometry data were collected by Electrospray ion sources in the positive mode, and the collection range was 100–1700 *m*/*z*.

## Data Availability

The data presented in this study are available in the article.

## Sample Availability

Samples of the compounds GA and GAA are available from the authors.

## Appendix A

The appendix provides data supplemental to the main text.

**Table A1:** Primers used in GAO gene cloning.

Primer	Primer Sequence
*Xs*GAO1F	ATGGAAGTCTCCCTCACCACTTC
*Xs*GAO1R	TTAAAAACTTGGTACCAATATCAACCC
*Xs*GAO2F	ATGGAACTCCATTTTCCCAC
*Xs*GAO2R	TCCAAATATCACTATCCTTCG
M13F	CAGGAAACAGCTATGAC
M13R	GTAAAACGACGGCCAGT

**Table A2:** Primers used in RT-PCR, qRT-PCR, and expression in yeast.

Primer	Primer Sequence
Q*Xs*GAO1F	CTAATAAGGTGTCCGAGAG
Q*Xs*GAO1R	GGCAGGTCTTGAATATCT
Q*Xs*GAO2F	TCTCAACCATAGTAATCTCA
Q*Xs*GAO2R	CGATGTCTGTGTAATTGTAT
ActinF	TACTACAACGGCAGAACGGGAAA
ActinR	TCATAGACGGCTGGAACAAAACC
GFP-*Xs*GAO1F	acgggggactcttgaccatggATGGAAGTCTCCCTCACCACTTC
GFP-*Xs*GAO1R	gcccttgctcaccatactagtAAAACTTGGTACCAATATCAACCCA
GFP-*Xs*GAO2F	acgggggactcttgaccatggATGGAACTCCATTTTCCCACC
GFP-*Xs*GAO2R	gcccttgctcaccatactagtCATTGTGTTGTAAGGTGTTGGGA
*Ls*GASF	ATGGCAGCAGTTGACACTAATG
*Ls*GASR	TTACATGGATACAGAACCAAC
*Ls*GAOF	ATGGAGCTTTCAATAACCACC
*Ls*GAOR	CTAAAAACTCGGTACGAGTAACAAC
pYeDP60-*Xs*GAO1F	acacactaaattaccggatccATGGAAGTCTCCCTCACCACTTC
pYeDP60-*Xs*GAO1R	gggagatcccccgcggaattcTTAAAAACTTGGTACCAATATCAACCC
pYeDP60-*Xs*GAO2F	acacactaaattaccggatccATGGAACTCCATTTTCCCACC
pYeDP60-*Xs*GAO2R	gggagatcccccgcggaattcTCACATTGTGTTGTAAGGTGTTGG
pYeDP60-*Ls*GAOF	acacactaaattaccggatccATGGAGCTTTCAATAACCACCTCC
pYeDP60-*Ls*GAOR	gggagatcccccgcggaattcCTAAAAACTCGGTACGAGTAACAACTC

**Table A3:** Reference sequences used for phylogeny construction.

Name	Species	Gene Bank Accession No.
*CcVs*GAO1	*Cynara cardunculus* var. scolymus	AIA09035.1
*CcVs*GAO2		XP_024977750.1
*CcVs*GAO3		AIA09037.1
*CcVs*-GAOlike		XP_024977969.1
*Tp*GAO	*Tanacetum parthenium*	AHN62855.1
*Ls*GAO1	*Lactuca sativa*	XP_023734551.1
*Ls*GAO2		ADF32078.1
*Ls*GAO3		AIX97103.1
*Bs*GAO	*Barnadesia spinosa*	ADF43083.1
*Ha*GAO	*Helianthus annuus*	ADF43082.1
*Sc*GAO	*Saussurea costus*	ADF43081.1
*Ci*GAO	*Cichorium intybus*	ADF43080.1
*Tc*GAO	*Tanacetum cinerariifolium*	AGO03789.1
*Ce*GAO1	*Cichorium endivia*	AZI95573.1
*Ce*GAO2		AZI95575.1
*Ce*GAO3		AZI95574.1
*OsJG*GAO	*Oryza sativa* Japonica Group	XP_015624875.1
